# Review of Synthesis and Separation Application of Metal-Organic Framework-Based Mixed-Matrix Membranes

**DOI:** 10.3390/polym15081950

**Published:** 2023-04-20

**Authors:** Lu Wang, Jingzhe Huang, Zonghao Li, Zhiwu Han, Jianhua Fan

**Affiliations:** 1 College of Food Science and Engineering, Jilin University, Changchun 130062, China; 2 Research Institute, Jilin University, Yibin 644500, China; 3 Key Laboratory of Bionics Engineering of Ministry of Education, Jilin University, Changchun 130022, China; 4 School of Mechanical and Aerospace Engineering, Jilin University, Changchun 130025, China

**Keywords:** metal-organic framework, MOF membranes, mixed-matrix MOF membranes, MOF membrane application

## Abstract

Metal-organic frameworks (MOFs) are porous crystalline materials assembled from organic ligands and metallic secondary building blocks. Their special structural composition gives them the advantages of high porosity, high specific surface area, adjustable pore size, and good stability. MOF membranes and MOF-based mixed-matrix membranes prepared from MOF crystals have ultra-high porosity, uniform pore size, excellent adsorption properties, high selectivity, and high throughput, which contribute to their being widely used in separation fields. This review summarizes the synthesis methods of MOF membranes, including in situ growth, secondary growth, and electrochemical methods. Mixed-matrix membranes composed of Zeolite Imidazolate Frameworks (ZIF), University of Oslo (UIO), and Materials of Institute Lavoisier (MIL) frameworks are introduced. In addition, the main applications of MOF membranes in lithium–sulfur battery separators, wastewater purification, seawater desalination, and gas separation are reviewed. Finally, we review the development prospects of MOF membranes for the large-scale application of MOF membranes in factories.

## 1. Introduction

With the continuous development of membrane science, membrane technology has achieved tremendous development in field concerning chemicals, petroleum, energy, aerospace, food, and environmental protection. According to the material of the membrane, membranes can be roughly divided into two categories: organic membranes and inorganic membranes. Organic membranes mainly include polyethersulfone and polyvinylidene fluoride membranes, with shortcomings that include a relatively short service life, poor thermal stability, and low selectivity, which limit the application of polymer membranes in the field of membrane separation [1]. Meanwhile, inorganic membranes mainly include ceramic membranes and molecular sieve membranes, which are not easy to prepare and are fragile [2]. Therefore, new membrane materials need to be developed to meet higher demands regarding separation performance.

Metal-organic framework (MOF) materials have been favored by researchers since they were first prepared in 1995 [3]. More recently, MOFs are considered to be one of the most promising emerging porous materials. MOFs are composed of metal ions and metal clusters linked with organic ligands. This structure, assembled by strong coordination bonds, gives MOFs an open, crystalline framework and enables the porosity of MOFs to be adjusted via the ratio of material compositions [4,5]. Since MOFs have the advantages of high specific surface area and large porosity, they are suitable for various applications, such as gas adsorption and energy storage [6]. Therefore, MOF membranes prepared from MOFs exhibit more excellent performance than conventional membranes in the field of adsorption and separation due to their unique advantages of high selectivity and permeability.

Currently, MOFs can be divided into two categories according to the composition of the membrane. One consists of MOF membranes composed of MOFs only, and the other concerns mixed-matrix membranes (MMMs) composed of both MOF materials and polymer materials. These two kinds of membranes have different characteristics. MOF membranes can be designed with suitable pore sizes according to different separation requirements, while MMMs have the advantages of a filling phase and dispersed phase, and a simple preparation process. Polymer membranes present a trade-off between permeability and selectivity in the separation process, such as Robeson’s upper bound. In contrast, the addition of MOF particles to a polymer can improve both membrane selectivity and permeability [7]. With the unique microporous structure of MOF materials, MOF-based membranes have great application prospects in the fields of separation and purification. Depending on the system of application, separation can be divided into solid–liquid separation, liquid separation, and gas separation. The main applications of solid separation are battery separators [8,9], seawater desalination [10,11], and wastewater separation [12,13,14,15,16].

This review will focus on several methods for the preparation of MOF membranes. In addition to the conventional methods, several new methods for preparing MOF membranes are introduced in this review, such as the freezing-assisted in situ growth method and the electrochemical-assisted in situ growth method. Several MOF-based hybrid matrix films are presented from the perspective of filling different MOF materials, which include Zeolite Imidazolate Frameworks (ZIFs), University of Oslo (UIO), and Materials of Institute Lavoisier (MIL) frameworks. Specific applications of MOF membranes, including on lithium–sulfur battery separators, wastewater purification, seawater desalination, and gas separation, are also presented.

## 2. Preparation of MOF Membrane

**Figure 1:**
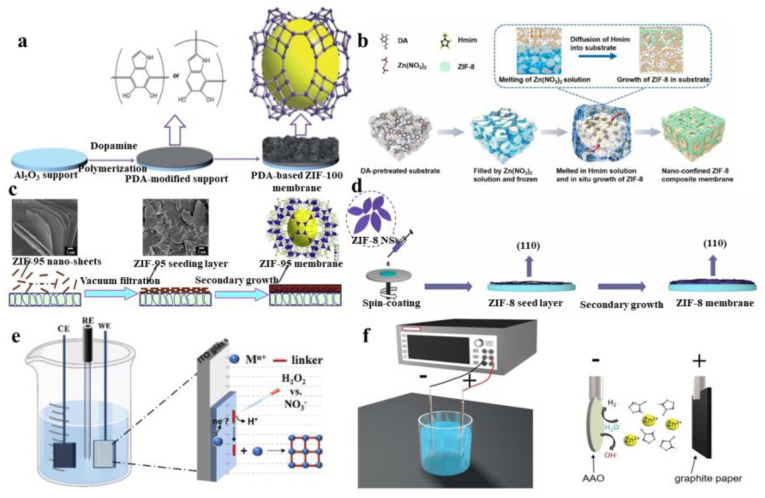
Various methods of preparing MOF membrane. (**a**) Preparation of ZIF-100 using the in situ growth method. (**b**) Preparation of ZIF-8 membrane using the freezing-assisted in situ growth method. (**c**) Preparation of ZIF-95 membrane using the secondary growth method. (**d**) Preparation of ZIF-8 membrane using the secondary growth method at a low temperature. (**e**) Preparation of MOF membranes by cathodic deposition. (**f**) Preparation of ZIF-8 membranes by aqueous cathodic deposition.

Commonly used methods of preparation of MOF membranes include the in situ growth method [17], secondary growth method [18], and electrochemical methods [19]. Each method has its unique advantages: for example, the in situ growth method can obtain high-quality single crystals and the preparation method is simple; the secondary growth method can control the final orientation of the membrane and obtain a dense membrane; and the electrochemical method requires a lower synthesis temperature (Figure 1) [20,21,22,23,24,25].

### 2.1. In Situ Growth Method

The in situ growth method involves directly contacting the reaction solution with the substrate and growing the MOF membrane on the surface of the substrate. According to the different substrates, the in situ growth method can be mainly divided into the direct growth method and the modified substrate method.

#### 2.1.1. Direct Growth Method

The direct growth method involves growing MOF membrane using unmodified substrates. The heat required for this preparation method is generally provided by the hydrothermal or solvothermal reaction. In addition, the substrate is directly in contact with the precursor sol or solution, and MOF crystals are directly nucleated and grown on the substrate. A previous study confirmed that MOF-5 membranes with good continuity could be successfully prepared on the surface of unmodified alumina substrates via the hydrothermal method and solvothermal method [17]. In addition, porous titanium dioxide was used as a substrate in one previous study. The precursor solution consisted of a mixture of methanolic solution of zinc chloride, 2-methylimidazole, and sodium formate. The titanium dioxide substrate was immersed in the precursor solution for 20 min and then heated at 100 °C for 4 h. ZIF-8 membranes were obtained after cooling and washing repeatedly with methanol [26]. Although the direct synthesis method makes it very simple to prepare MOF membranes, it is not commonly used due to the lack of nucleation sites on the substrate, which cannot guarantee tight bonding between the crystal and the substrate [27].

#### 2.1.2. Modified Substrates Method

With the deepening of research, some researchers have found that the loose binding of MOF to a substrate would make the prepared membrane discontinuous. At present, the modification of the substrate is generally considered to be a more effective method to improve the bonding of the substrate to the MOF membrane. Commonly used modification methods include organic modification and inorganic compound modification [27].

Polydopamine (PDA) has often been used as a modifying material in organic modification methods. The binding effect of PDA is effective in increasing the compatibility and interaction between the MOF filler and the substrate [28]. In addition, membranes prepared using PDA-modified substrates had better continuity. ZIF-100 has a complex crystal structure with a high affinity for CO_2_ and high thermal stability. During the preparation of ZIF-100 membranes, if ZIF-100 was directly synthesized on an α-alumina substrate, it was difficult to obtain a continuous ZIF-100 membrane. A previous study demonstrated that by using PDA-modified α-alumina as the substrate, placing it in an autoclave filled with a synthetic solution, inducing the thermal reaction of dissolution, and washing with DMF many times, a continuous ZIF-100 membrane with much denser and thicker materials could be obtained (Figure 1a) [20]. In addition, some researchers have proposed a method that can be used to fabricate a ZIF-8 membrane using Zn^2+^-doped PDA-modified substrates. ZIF-8 is a widely studied ZIF material, which is probably because of its stability and flexible framework. It is constructed from Zn^2+^ and 2-methylimidazole with sodalite (SOD) topology [29]. For this process, PDA and NaIO_4_ were dissolved in a buffer solution as a modification solution of substrates, and the addition of NaIO_4_ could effectively accelerate the precipitation of PDA on substrates. The crystallinity and the degree of order of prepared ZIF-8 membranes were higher compared with conventional ZIF membranes, and the synthesis time was significantly shortened due to the effect of Zn^2+^ [30].

When inorganic compounds were used to modify the substrate, the same inorganic compound as the metal contained in the MOF membrane was generally selected, because the inorganic compound could directly participate in the reaction as a metal source. The previous study found that zinc-based sol was applied to the substrate to obtain ZnO, and then a ZIF membrane with good thermal stability and good separation performance could be obtained. In this method, the ZnO nanorods layer on the substrate had multiple functions, including providing a metal source, nucleation sites for the growth of ZIF nanosheet membrane, and anchoring sites between membrane and substrate, which were conducive to the formation of the stable and continuous orientation of the nanosheet membrane [31]. In addition, a previous study confirmed the efficient preparation of ZIF membranes using zinc oxide as the metal source and ammonium oxide as the modifier. The addition of ammonium oxide can effectively control the orientation of the membrane, while the self-transformation of ZnO and the auxiliary action of ammonia can achieve ultrathin-ZIF membranes (50 nm) with good permeability and selectivity to H_2_ [32]. Therefore, the use of the same metal oxides as the modification materials can make the combination of the membrane and the substrate occur more tightly and produce denser membranes, providing an effective novel method for the preparation of MOF membranes.

The modification of substrates by both organic molecules and inorganic molecules results in a tighter binding between the membrane and the substrate. The difference is that in the former, organic molecules can form substrates with nucleation sites and effectively enhance the binding force with MOF, while the latter penetrates the MOF membrane through the direct participation of the metal source in the reaction. In addition, the nanostructure of inorganic molecules increases the surface area of the substrate, provides initial attachment sites for MOFs, and makes the membrane more inclined to grow on the substrate surface [27].

In addition to some of the traditional in situ growth methods mentioned above, the use of other techniques in combination with them has been gradually emerging. An in situ growth method using freeze-assisted growth has been developed. After the substrate was immersed in the metal solution, it was frozen with liquid nitrogen and finally immersed in a 2-methylimi-dazole (Hmim) solution. After freezing, the particles of ZIF-8 grow in the pores of the substrate, which eliminates the need for a dense separation layer on the membrane surface. This method proposes a good way of overcoming the disadvantages of the ZIF-8 membrane in preparation (Figure 1b) [21]. In addition to using freeze-assisted methods, other researchers have used electrochemically assisted in situ growth. For this method, the solvent was replaced by metal plates and the metal ions required for the reaction were supplied by said metal plates. Metal ions were continuously deposited on the surface of the substrate, and eventually, a continuous film was obtained [33].

### 2.2. Secondary Growth Method

Most methods aimed at the synthesis of MOF membranes consist of immersing the substrate in a mixed solution of metal ions and organic ligands so that the MOF membrane may grow directly on the substrate. However, direct growth can lead to the heterogeneous nucleation of the prepared MOF substrate, resulting in membrane defects. The secondary growth method makes it easier to control the final orientation of the membrane and can produce a more dense and continuous MOF membrane [34].

The secondary growth method first requires seeds to be sown on the substrate, and after the seed layer is formed, the seed layer grows a continuous MOF membrane after heating. Some researchers have used metal oxide induction to prepare the seed layer. ZIF-95 has high thermal stability, retains its structural stability at 500 °C, and also has a huge cavity and narrow pore size. Some researchers have successfully prepared ZIF-95 nanosheets as seeds. The seeds were wrapped in the substrate and then crystallized in an air oven. A ZIF-95 membrane was obtained after being cleaned with methanol (Figure 1c) [22]. During the process, a layer of 3-aminopropyltriethoxysilane (APTEs) could be deposited on the substrate using the mechanism of the reaction between APTEs and alumina substrate. The substrate was repeatedly immersed in the mixed solution of H_2_OEt-IPA and Cu(NO_3_)_2_ to effectively obtain the seed layer. After secondary growth, the Kgm-OEt membrane could be obtained. During this process, membranes grown on the APETs-treated substrates were continuous and defect-free and had an orientation suitable for gas transmission [18]. Some researchers attempted to perform secondary growth at low temperatures. After the seed layer was prepared, the precursor solution was prepared in an ice-water bath environment, after which the seed layer was placed in a low-temperature reaction for secondary growth. In this preparation process, low temperatures can effectively eliminate the gap (Figure 1d) [23].

### 2.3. Electrochemical Method

Compared with other methods, the electrochemical method can be synthesized at room temperature with low energy consumption, thus meaning it is regarded as a green synthesis method. In addition, the reaction time is short and the required equipment is simple and convenient [35]. Recently, electrochemical methods have mainly included anode synthesis and cathode synthesis [36].

The anode precipitation method mainly uses a high, positive voltage to dissolve the metal in the anode, and the generated metal ions react with the ligand to obtain the MOF membrane at the anode [37]. During the preparation of MOF membranes using electrochemical methods, if the ions in the MOF membrane are high-valent cations, they may require a high reaction temperature. In a recent study, researchers used a new high-temperature and high-pressure battery, which could effectively prepare MOF membranes composed of high-valent cations. The solution used for this method was environmentally friendly and non-corrosive [19].

When the MOF membrane is prepared by the cathodic deposition method, the metal source for cathodic deposition is generally obtained by adding metal salts. However, when preparing MOF films by cathodic precipitation, impurities may be generated, and even MOF particles cannot be formed. In response to the above problems, the researchers proposed a method that used hydrogen peroxide to prepare MOF membranes. Hydrogen peroxide was oxidized to a superoxide in the reaction to deprotonate the OH^−^ ligand, which could effectively inhibit the co-precipitation of metals and produce a high-purity MOF membrane (Figure 1e) [24]. In addition, the electrochemical methods used to prepare MOF membranes cause damage to the environment due to the use of various solvents. Since the researchers presented a cathodic precipitation method using water as the only solvent, the resulting membrane was prepared without the use of a supporting electrolyte, yielding a ZIF-8 membrane with low defect density after only one hour. This presented a simple and pollution-free method for the preparation of MOF membranes (Figure 1f) [25].

The difference between the cathode synthesis method and the anode synthesis method is that the metal ions in the cathode synthesis method are not formed by the dissolution of the electrode, but metal ions are added, and then the OH- generated at the cathode deprotonates the ligand, making it self-assemble with metal ions on the electrode surface to form MOFs membranes. The membranes of the anodic deposition method form more easily on the substrate, while the membranes of the cathodic deposition method form more easily on the electrodes [38]. The characteristics of the above three synthesis methods are shown in Table 1

## 3. Mixed-Matrix Membranes

During the use of filtration membranes, the balance between selectivity and permeability limits their application. Mixed-matrix membranes (MMMs) can solve this problem very well and can effectively improve the performance of the membrane by adding different filler materials. By improving the compatibility of the filler material with the polymer, the performance of the membrane can be improved. Therefore, the key to the preparation of mixed-matrix membranes is the selection of the correct combination of filler and dispersed phases [40]. MOFs are suitable as filling materials for MMMs due to their adjustable pore sizes. MOFs are commonly used as filler materials, (including Zeolite Imidazolate Frameworks, ZIF, University of Oslo, UIO, and Materials of Institute Lavoisier frameworks, MILs); their advantages and disadvantages are shown in Table 2.

### 3.1. Zeolite Imidazolate Frameworks

Zeolite Imidazolate Frameworks (ZIFs) molecular sieve imidazolate framework is a type of MOF. ZIF is a porous coordination polymer with homogeneous micropores and large pores in which divalent metal ions are connected to four non-derivative ligands. The common cations in ZIF are Zn(II) or Co(II) [50]. Due to their excellent thermal stability, chemical stability and gas adsorption capacity, ZIFs are favored by researchers [51]. ZIF-8 is a suitable nanoparticle to fill polymers in various ZIF crystals. In order to improve the selectivity of the prepared mixed-matrix membranes, researchers usually add some materials with excellent properties, such as graphene oxide (GO), to the casting solution. Some researchers have successfully prepared ZIF-8/GO composites by nucleating ZIF-8 grown on the surface of graphene oxide and used it as a filler phase to prepare hybrid matrix films. During the separation process, the MOF in the composite acted to enhance the CO_2_ affinity of the membrane, and GO acted to limit the diffusion of macromolecules, both of which increase the selectivity of the membrane for CO_2_/CH_4_ by 61% [52].

**Figure 2:**
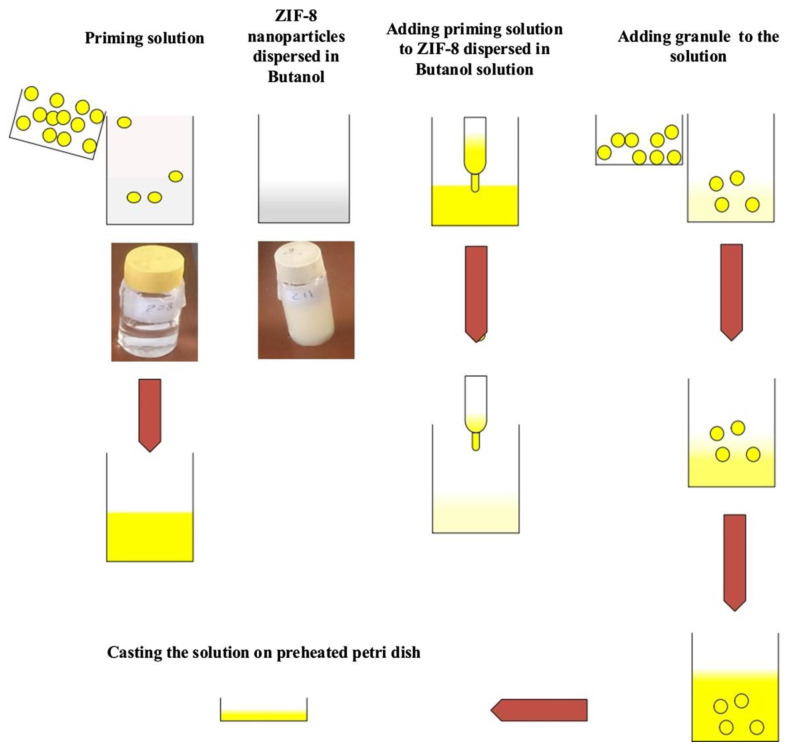
The preparation method used for MMMs.

During the process of membrane preparation, if metal-organic frameworks, such as ZIF, are directly added to polymers for the preparation of mixed-matrix membranes, non-selective interfacial voids and insufficient adhesion may occur. One previous study confirmed that adding ionic liquids can effectively solve the above problems [53]. MOF particles are modified by ionic liquids, and the modified MOF particles were used to fill polymers to produce mixed-matrix membranes with efficient CO_2_ adsorption capacities (Figure 2) [54]. In addition, to explore the effect of different ionic liquids on mixed-matrix membranes, ZIF-8 nanoparticles were modified by selecting different loadings of various types of ionic liquids and using them as filling materials. The study found that filling ionic liquids with high affinity to be used as modification materials could improve the CO_2_ selectivity of the prepared mixed-matrix membranes, but excessive loading had little effect on the performance of the mixed-matrix membranes [55].

The addition of MOF particles improved the CO_2_ permeability of the mixed-matrix membrane, but at the same time decreased its CO_2_ selectivity, which might be due to the poor compatibility between the MOF particles and the membrane. It was found that amine-functionalized ZIF-8 could effectively improve the permeability and selectivity of the membrane, which was due to chemical interactions and the effect of the controlled fence combination that enhanced the selectivity of the membrane for CO_2_ [56]_._ In addition, it was found that the crystal structure and thermal stability of the synthesized NH-ZIF-8 were identical to those of the pure ZIF-8 crystals. By adding it to the polymer Pebax, the compatibility of ZIF with Pebax was improved, and the modification of amino groups can also improve the affinity of ZIF-8 for CO_2_. Therefore, this amino-modified membrane has higher CO_2_ permeability and separation performance [57]. The hydrogen bond between the ammoniated ZIF-8 and the polymer was strengthened, which also increased the hydrophilicity of the membrane [58].

**Figure 3:**
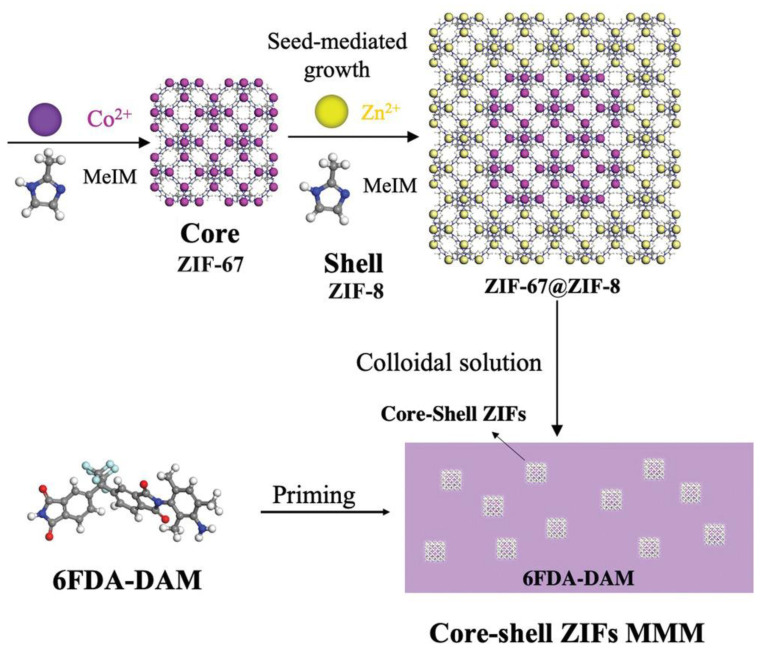
The preparation process of mixed-matrix membranes with core-shell ZIF-67@ZIF-8.

In addition to using the pure ZIF-8 crystals described above or modified ZIF-8 crystals as the dispersed phase to prepare mixed-matrix membranes, a new method using core-shell structures as filler materials has been proposed. By growing one MOF on the surface of another MOF ionic surface, a core-shell structure MOF crystal is obtained, which has the excellent properties of both a core structure and a shell structure. For example, ZIF-67 and ZIF-8 have the same topology, which makes them easy to combine into a core-shell structure, enabling the preparation of highly permeable mixed-matrix membranes with ZIF-67@ZIF-8 core-shell structures (Figure 3) [59]. Because the surface of ZIF-67 crystals grew unevenly in the study that attempted this, the core-shell nanoparticles had higher specific surface areas and rougher surfaces, which made the connection between crystals and polymers much stronger, and the mechanical properties of the mixed-matrix membranes were also significantly improved. In addition, it was found that in the process of preparing the ZIF-8@ZIF-67 core-shell structure, since the solvothermal method was difficult to control during the reaction process, the precise layered core-shell structure could not be obtained, so it was not suitable to use the solvothermal method. However, the core-shell structure was prepared by the self-assembly method, which could successfully solve this problem and prepare a mixed-matrix membrane with high performance. In particular, the mixed-matrix membranes prepared with ZIF-8@ZIF-67 core-shell structures exhibited nearly twice the performance in terms of H_2_/CO_2_ separation performance compared with membranes prepared from other MOF materials [60].

### 3.2. University of Oslo

University of Oslo (UIO) is a type of MOF material. It was usually constructed from Zr4^+^ and dicarboxylic acid ligands. UIO-MOF has many active binding sites. Compared with other MOF particles, UIO-MOF has good thermal and chemical stability as well as strong hydrodynamic properties [44,47]. Common UIO-MOFs include UIO-66 and UIO-67. During the preparation of mixed-matrix membranes, to enhance the interaction between polymers and MOFs, MOFs are often modified to have some specific functional groups. When using UIO nanoparticles to prepare mixed-matrix membranes, -NH_2_ is often used as a modification group. When UIO-66-NH_2_ is used to fill the polymer, hydrogen bonds form between the two, making the membrane more compact. Moreover, the UIO-66-NH_2_ in the polymer has also been shown to improve the permeability of the membrane because of the shortcuts for gas provided by UIO-66-NH_2_ [61]. The amino groups in the modified nanoparticles can react with functional groups in the matrix or other modified materials, improving the interaction between the MOF and the polymers. By modifying UIO-66 with NH_2_-BDC, imidazole-2-carbaldehyde (ICA) is grafted onto the nanoparticles through the reaction of amino groups with ICA. In this mixed-matrix membrane, the introduction of ICA in one study led to an increase in the number of nitrogen atoms on the membrane surface, which facilitated the incorporation of CO_2_ [62]. In addition to amination, UIO-66-NH_2_ can be further modified by using bromomethylated poly (BPPO) as a coupling agent to react with the -NH_2_ group, which could enhance the interaction between the MOF and the polymer, which in turn has been shown to improve the thermal stability and gas separation performance of the mixed-matrix membrane [61].

In terms of modifying UIO nanoparticles, in addition to using -NH_2_ modification, other materials can be used for modification, such as branched polyethyleneimine (PEI), polydimethylsiloxane (PDMS), and azobenzene groups. The multiple primary amine sites on PEI enables it to promote the adsorption of CO_2_. Therefore, PEI has often been used to modify porous materials to improve the adsorption performance of materials for CO_2_. 4,4′-(hexafluoroisopropylidene) diphthalic anhydride-4,4′-diphenylamine (6FDA-ODA) was selected as the polymer matrix to successfully prepare a mixed-matrix membrane loaded with UIO-66-PEI particles with high separation efficiency [63]. PDMS is also a polymer commonly used in gas separation membranes, but when used with MOF particles, it causes pore blockage, thus reducing membrane permeability. In a recent study, researchers tried to use covalent grafting to improve the dispersion of MOF particles in polymers, which would reduce the clogging of PDMS and MOF. The gas permeability of the membrane was improved without decreasing the selectivity, and there was no obvious defect on the surface [64]. When Azo-UiO-66 was used to fill the polymer, the permeability of the membrane changed depending on the presence or absence of UV irradiation, which was due to the presence of the azobenzene guest molecule. A mixed-matrix membrane using Azo-UiO-66 as a filler material was reported. The Azobenzene group in Azo-UiO-66 placed it in a nitrogen-rich environment, thus reducing its nitrogen affinity and improving CO_2_/N_2_ selectivity [65]. Different combinations of UIO-66 hybrid matrix films are shown in Table 3.

### 3.3. Materials of Institute Lavoisier Frameworks

MIL has also been a popular MOF material over recent years. The common MIL today usually consists of carboxylates and cations. These cations are all trivalent, such as iron, chromium, and aluminum [49]. MIL is a MOF with high porosity. It also has high thermal and chemical stability. Currently, most MIL-based mixed-matrix membranes are applied in the direction of gas separation. For example, MIL-68(Al) has a rich void structure and is rich in hydroxyl groups in the pore channels, which makes it highly adsorbable to CO_2_. Using this property, some researchers have prepared hybrid mechanism membranes with high CO_2_/CH_4_ separation performance, with CO_2_/CH_4_ selectivity values of up to 79 [48]. In order to overcome the compatibility problem of fillers in mixed-matrix membranes, some researchers have proposed the preparation of gel mixed-matrix membranes, and during the preparation process, it was found that the addition of MIL-101(Cr) could improve the mechanical properties of the membranes as well as the gas separation properties compared with conventional gel membranes. In the subsequent study, it was found that further modification of MIL-101(Cr) with tripropionin could promote the dispersion of MIL-101(Cr), thus further improving the separation performance [70]. Some researchers have attempted to synthesize MIL-101(Al) in carbon nanotubes (CNTs). This new composite material could improve the selective adsorption of CNTs. By growing MIL-101(Al) in situ in CNTs, active sites were successfully introduced. The permeability of the prepared membranes was also improved by using MOF-modified CNTs to fill the polymer, enhancing the permeability to 2.5 times that of the pure polymer membrane.

## 4. Application

**Figure 4:**
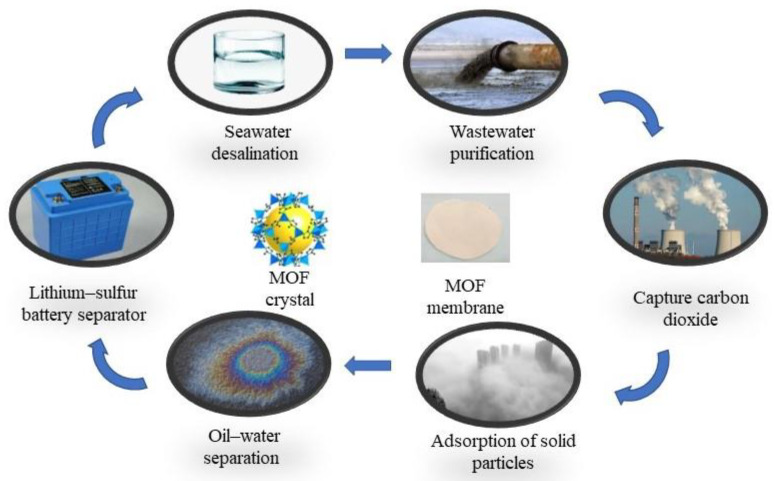
MOF crystal, MOF membrane, and applications of MOF membranes.

Due to their excellent separation performance, MOF membranes have been widely used across various fields in recent years, such as the desalination of seawater, purification of industrial wastewater, and separation of gases (Figure 4) [51,55]. The application of MOF in different applicable systems will be described in detail below.

### 4.1. Application in Solid and Liquid Phases

In solid–liquid systems, MOFs are mainly used in separators of lithium–sulfur batteries, the purification of wastewater, and the desalination of seawater, as shown in Table 4.

#### 4.1.1. Battery Separator

The increase in human demand for energy and the polluting impact fossil energy on the environment has stimulated the development of new energy. Due to the abundant resources, low cost, and high theoretical specific energy of lithium–sulfur batteries, they are gradually attracting much attention. However, there are many problems with the applications, which limit the development of lithium–sulfur batteries. For example, soluble long-chain Li_2_S_n_ is generated during the charging and discharging process of the battery [71]. This long-chain substance is dissolved in the electrolyte and produces a shuttle effect. In addition, during the charging and discharging process, due to the uneven deposition of lithium, the formation of lithium dendrites may pierce the battery separator and cause the battery to short circuit. These problems severely affect the life and performance of batteries. Therefore, the separator used in lithium–sulfur batteries not only needs to have certain flexural resistance and conductivity but also needs to be able to effectively inhibit the shuttle effect of polysulfides and quickly capture the active material of the positive electrode. Therefore, MOFs with highly ordered pores, adjustable porosity, and large specific surface areas are very suitable as ionic sieve materials for the preparation of separators in lithium–sulfur batteries [72,73].

In recent studies, some scholars have used Mn-BTC MOF, PVDF (polyvinylidene difluoride), and acetone as coating solutions to coat the separators of lithium–sulfur batteries. Due to the large specific surface areas of the MOF particles, the electrolyte can penetrate the voids, which improves the conductivity of the separator, with conductivity improving from 5.1 × 10^−4^ S·cm^−1^ with uncoated MOF to 5.6 × 10^−4^ S·cm^−1^ with coated MOF. In addition, the shuttle effect of polysulfides has been shown to be suppressed by the ionic force generated by COO- in the coating solution, which could effectively suppress the shuttle effect of polysulfides, thereby improving the charge cycle performance of lithium–sulfur batteries. The batteries using this separator had a good cycle life, with only a 3.86% loss of battery discharge capacity after 10 cycles [8].

In addition, polyolefin membranes are often used as battery separators in lithium–sulfur batteries. Due to the low melting point of polyolefin membranes and high operating temperatures during charging and discharging, there is a great safety problem. To overcome this problem, in addition to coating different materials on the original separator to modify it, choosing a suitable alternative separator material to improve the thermal stability of the separator has become a research hotspot regarding lithium–sulfur batteries. One previous study confirmed that the use of MOF materials could effectively produce high-performance nanofiber membranes loaded with ZIF-67 nanoparticles and Cu-BTC nanoparticles via electrospinning. Because of the addition of MOF particles, the porosity of the membrane increased, which was beneficial to improving the penetration and diffusion of electrolytes, thereby effectively inhibiting the “shuttle effect” and reducing the formation of dendrites. Additionally, during the charging and discharging process of the battery, the decomposition of MOF generated carbon particles, which effectively improved the stability of the membranes. In addition, the fluorinated emulsion in the casting solution reacted with the lithium salt in the electrolyte. The addition of fluorinated emulsion and MOF particles could therefore reduce the fiber diameter of the membrane, thereby effectively improving the electrolyte affinity of the membranes [9].

#### 4.1.2. Seawater Desalination

The process of removing salt ions from seawater is called seawater desalination. Although the application of MOF membranes in seawater desalination is still in the development stage, it has shown great potential in desalination, nanofiltration, and other filtration processes involved in seawater treatment [74]. In particular, the new thin-film nanocomposite (TFN) membrane has limited further wide application due to its high cost, poor stability, and easy leaching of nanoparticles during use [75]. Some researchers tried using MOF particles as filler particles for TNF reverse osmosis (RO) membranes. MOF particles were deposited on the film surface through an atomization-assisted pre-deposition method, which reduces the time for MOF to be deposited on the membrane. It also improved the chlorine resistance of the membrane, and the salt rejection was still higher than 93% after being subjected to high-concentration NaClO treatment. When this RO membrane was used in seawater desalination experiments, a membrane salt rejection of 99.2% and a water flux of 40 L·m^−2^·h^−1^ were found [76].

Although reverse osmosis (RO) technology is considered to be a promising desalination method, the high energy consumption limits its application in the filtration environment. Pervaporation (PV) technology can not only reduce the energy consumption in the desalination process but also produce high-purity water compared to RO. However, during the pervaporation process, the filter membrane was found to be unstable [77]. To solve this problem, a highly stable and highly selective UIO-66 thin membrane was prepared by using a secondary growth method to induce the seed layer of UIO-66 with titanium dioxide with a salt rejection rate of 99.9% and a water flux of 37.4 L·m^−2^·h^−1^. The membrane not only performs well in high-salt and low-salt solutions but also has good stability even under extreme conditions [11].

#### 4.1.3. Wastewater Purification

Water is one of the most important resources for human beings. With the recent surge in population, the freshwater resources of the earth are gradually decreasing, and the number of people in a state of water shortage continues to rise. In addition to the desalination mentioned in the above section, the purification of domestic and industrial wastewater has gradually become an effective method used to alleviate the current water shortage. The purification principle of wastewater is similar to that of seawater desalination, and the main purpose is to separate impurities such as heavy metals and wastes from wastewater [10]. In the purification of wastewater, the use of adsorbents such as activated carbon to remove pollutants is considered a relatively simple method; however, these adsorbents have poor adsorption efficiency and low selectivity, which cannot meet the expectations of adsorbents [78]. Research has found that MOF materials have excellent adsorption performance for heavy metals, which make them a potential heavy metal adsorbent. Therefore, MOF membranes could effectively purify wastewater [79].

Mercury can be accumulated in the human body through water and food, posing a serious threat to human health [80]. One previous study confirmed that a MOF membrane (UIO-66-S), which was prepared using ZrCl_4_, DMF, and other reagents, could effectively adsorb mercury in wastewater. The membrane presented excellent mercury removal efficiency and could remove more than 80% of mercury in wastewater within 20 min. In addition, the membrane solved the problems of unstable MOF materials and difficult membrane regeneration during the purification process, so that the MOFs could present excellent stability and durability. After multiple purifications of wastewater, the mercury removal rate of the membrane could be maintained above 98% [12].

In addition to mercury, a large amount of lead-containing wastewater is also produced in industrial production, especially in battery industries. Lead is classified as a pollutant and is very harmful to humans and plants. Therefore, the treatment of lead-containing wastewater has become the primary problem facing current wastewater treatment [81]. Some researchers have combined UIO-66-NH_2_ with ceramic membranes to prepare a ceramic membrane loaded with modified MOF particles, which effectively removed lead from wastewater. Under optimal conditions, the maximum removal rate of lead by this membrane reached 1795.3 mg·g^−1^. In addition, the regeneration ability and anti-fouling ability of the membrane were also excellent, and the pure water flux after the cleaning was no different from that of the original membrane at 1174 L·m^−2^·h^−1^ [13].

Copper has many uses in everyday life, and it may enter water in different ways. Once copper is excessive in the human body, it can cause various diseases. Therefore, copper in industrial wastewater also needs to be removed [82]. A method of using Zr-MOF to fill ceramic membranes to achieve the adsorption of copper ions in wastewater has been proposed. This membrane can achieve a retention capacity of 988.2 mg·g^−1^ for copper ions under optimal conditions, at a pH of 6, a transmembrane pressure of 0.05 MPa, a crossflow speed of 4.5 m·s^−1^, and a temperature of 40 °C [14].

With the development of the textile industry, the water pollution problem posed by dyes also needs to be solved urgently. The application of the membrane method to treat dyes in wastewater is an environmentally friendly method. A method for preparing nanofiltration membranes with efficient removal of dyes has been developed. One study found that the gallic acid monohydrate (GA)/ZIF-67 membrane was prepared on the surface of polyimide (PI), then immersed the membrane in polypyrrole (Py) solution. Ammonium sulfate was then added as an oxidant to form hydrogen bonds and covalent bonds between GA and Py. The stability and the rejection rate of the membrane were effectively improved. Additionally, the addition of MOF increased the water channel of the selective layer, which greatly improved the water flux of the membrane, with rejection rates for rose bengal (RB), methyl blue (MB), Congo red (CR), and bromothymol blue (BTB) of 98.53%, 98.7%, 99.19%, and 80.2%, respectively [15].

In addition, with the rapid development of agriculture, many organic pollutants such as antibiotics can pollute water sources. Such organic pollutants pose a considerable threat to both the environmental and human health. Unlike the adsorption method to treat heavy metals, the photocatalytic method can degrade organic pollutants in water. When MOF treats wastewater, it is usually separated from the suspension, which is a time-consuming process. The preparation of MOF membranes with photocatalytic properties can effectively avoid this process. A nanofiber hybrid membrane carrying NH2-MIL-125 particles has been used to remove dyes from wastewater. This hybrid membrane can remove up to 56% and 60.5% of methylene blue(MB) and sodium fluorescein (SF), respectively [83].

### 4.2. Application in Liquid and Liquid Phase

**Figure 5:**
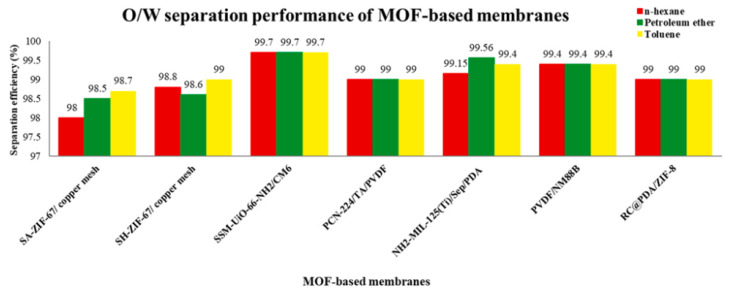
Performance of different MOF-based membranes in oil–water separation.

The main application of MOFs in liquid–liquid systems is oil–water separation. Traditional oil–water separation methods have obvious shortcomings in dealing with major pollution, such as large energy loss and low efficiency [84]. Therefore, the development of an oil–water separation membrane with good separation performance and low cost of use is in line with the existing major needs. With the rise of MOF materials, many researchers have realized that MOF materials with good structural characteristics are extremely suitable for the preparation of oil–water separation membranes. Figure 5 shows the performance of different MOF-based membranes in oil–water separation as summarized by a researcher [85]. 

#### 4.2.1. Treatment of Machining Oil Wastewater

The anti-fouling ability of a membrane can be significantly improved by introducing hydrophilic groups. Due to the hydrophilic groups on the surface of modified MOFs, these membranes have been shown to present high anti-fouling ability and stability during oil–water separation, as well as good separation performance after multiple cycles. A high-performance oil–water separation membrane was prepared by self-assembly of modified MOFs, and the retention rate of the pump oil reached up to 99.9% [86]. In addition, this study found that the oil–water separation efficiency of the membrane could also be effectively improved by using a metal-phenolic network (MPN) and MOF materials to build a multilayer membrane structure. Since the affinity provided by MPN made MOF particles disperse more uniformly, the water flux after water/pump oil emulsion separation could be as high as 6300 L/m^2^ h, and even after five cycles of use, the water flux was still as high as 5500 L/m^2^ h [84]. Additionally, some researchers have confirmed the formation of UIO-66 in graphene oxide (GO) nanosheets using a hydrothermal method and then modified it with polyacrylic acid (PAA) to prepare MOF membranes by vacuum-assisted self-assembly. The performance of the filtration membrane could be significantly improved, and the permeation flux of MOF membranes after pump oil–water separation reached 5067 L·m^−2^·h^−1^·bar^−1^, which was due to the increase in the distance between the membranes with the combination of GO and MOF. In addition, due to the introduction of abundant hydrophilic groups (carboxyl groups) on the membrane surface, the membrane had good anti-fouling properties, with the water flux of the membrane remaining above 80% of the original flux after three cycles of use [87]. An oil–water separation membrane with anti-fouling properties was prepared using melamine-modified UIO-66-NH2 (UiO-66-NH-Mlm) for the preparation of composite membranes. After modification, the number of -NH2 groups in the membrane increased, thus improving the hydrophilicity. Additionally, the contamination resistance of the membrane was improved, which was probably because the addition of MOF reduced the roughness of the membrane surface to retain more than 99% of the oil [88].

#### 4.2.2. Treatment of Edible Oil Wastewater

Kitchen garbage such as edible oil is often discharged into the sewer without treatment, causing huge amounts of damage to the environment [89]. At present, the most commonly used methods in the treatment of edible oil, such as adsorption, oxidation, and photocatalysis, all need to consume a lot of energy. Similar to the treatment of oily wastewater in industry, the membrane treatment method, with its low energy consumption and simple operation, has gradually become the first choice for treating edible oil [90].

The oil–water separation membrane was prepared with a nanofiber MOF selective layer built on a polyethersulfone (PES). The nanofibers carried in the separation membrane provided a flow channel for water molecules, so the membrane permeability was improved, producing a permeability coefficient of 46.4 L·m^−2^·h^−1^·bar^−1^. Additionally, due to the good photothermal properties of the nanofibers, the permeability of the membrane increased to 69.8 L·m^−2^·h^−1^·bar^−1^ in light, and the high charge of the nanofibers gives the membrane good separation performance. For the simulated waste edible oils produced from olive oil and sunflower oil, the rejection rates were 97.8% and 97.0%, respectively [90]. To obtain oil–water separation membranes with better separation performance, electrospinning was also a commonly used method. Several researchers prepared electrospun membranes with MIL-100 (Materials of Institute Lavoisier) for the separation of soybean oil/water mixtures. MIL-100 increased the surface roughness of the membrane, which made the membrane have good anti-oil fouling performance, and the oil-removal efficiency of the membrane remained above 99.0% even after five oil–water separation tests. Most importantly, compared with other oil–water separation membranes, the membrane also presented good filterability for food additives such as vanillin and amaranth, with a removal rate of over 99%. Therefore, the membrane presented a highly potential multifunctional filtration membrane [91].

#### 4.2.3. Treatment of Other Oil Wastewater

In addition to the above-mentioned oil wastewater, there are also some machining emulsified oil wastewaters, such as kerosene and lubricating oils. These oils will pollute the environment. In one study, ZIF-8 was etched with TA to obtain TA-ZIF-8, which was assembled with MXene on a cellulose acetate membrane to produce a composite membrane with excellent oil–water separation performance, with the retention rate of lubricating oil in particular reaching 98%. The water flux remained unchanged at 4432.8 L·m^−2^·h^−1^, even after eight cycles [92]. In addition, during the synthesis of MOF materials, due to the limitations of the method, the prepared MOFs were polyhedral, which limits their wide application in oil–water separation. The researchers proposed a novel method for preparing MOF materials to effectively solve the above problems. The copper mesh was soaked with NaOH solution and K_2_S_2_O_8_ to make Cu(OH)_2_ in the copper mesh, to grow MOFs in situ and successfully construct a layered structure. The ultra-wetting membrane prepared by this method required only a short time and had high separation efficiency and stability. Even after 20 cycles of use, the separation efficiency of the membrane for kerosene remained above 98.8% [93].

### 4.3. Application in Gas and Gas Phase

To address the excessive emissions of greenhouse gases, a common measure is to capture carbon dioxide from the exhaust gas of power plants [94]. Amine scrubbing is considered to be an effective method for capturing carbon dioxide, but it has gradually been replaced by new technologies due to its high price. MOFs have an excellent affinity for acidic gases and have suitable pore sizes, which enables them to capture CO_2_ effectively. They are gradually being applied to gas adsorption, such as to the treatment of the large amount of CO_2_ waste gas produced by burning fossil fuels in power plants [95,96].

**Figure 6:**
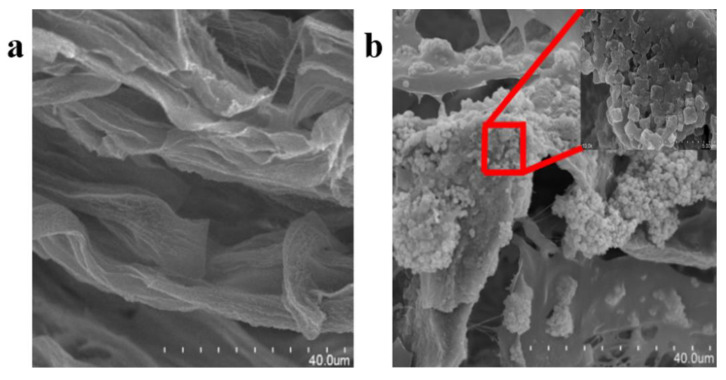
SEM images of (**a**) a blank PVA/CS hydrogel membrane without CA@ZIF-8, and (**b**) a PVA/CS/CA@ZIF-8 composite membrane.

A membrane-making method combining electrospinning and seed growth could effectively solve the problem of the low loading of MOF particles during electrospinning. Due to the increased loading capacity of HKUST-1 on the nanofiber membrane, the CO_2_ capture capacity has been shown to also be significantly improved. In addition, after using this membrane for multiple-cycle adsorption, the MOF membrane still had high CO_2_ adsorption performance, showing high cycle stability and low production costs. These advantages made this MOF membrane suitable for large-scale applications [97]. In addition, one previous study confirmed that a polyethylene-chitosan-hydrogel network as the base material (Figure 6a), and the network structure filled with carbonic anhydrase-modified MOF material (CA@ZIF-8) could produce a MOF membrane with excellent recyclability (Figure 6b). This porous structure, shown in Figure 6a, can improve the mass transfer of both matrix and product. Moreover, as shown in Figure 6b, CA@ZIF-8 can still keep its structure intact in this porous structure. The MOF membrane also had an excellent carbon dioxide capture capacity, which was 20 times higher than that of traditional carbonic anhydrase materials. Additionally, the MOF membrane exhibited good stability, and the activity of the membrane could be still maintained at half of the original value after 11 cycles. Moreover, the MOF membrane could be directly recovered after CO_2_ capture without complicated operations such as high-speed centrifugation, which reduces the cost of large-scale applications [98].

In addition to improving the cyclic stability of the membrane, capture cost can also be reduced by enhancing the permeability of the membrane to CO_2_. Commonly used methods include the modification of filled nanoparticles and the preparation of mixed-matrix membranes using multiple nanoparticles. In the previous study, filler particles were modified with PDA to enable them to have good viscosity, and the CO_2_ capture capacity of the membrane was greatly improved because PDA inhibited the transport of non-selective gases in the membrane. Especially, the selectivity of the prepared PDA/UIO-66 membrane in CO_2_/N_2_ was 51.6, which was more than twofold higher than that of other MOF-loaded membranes. Additionally, the PDA/UIO-66 membrane exhibited high stability even under humid conditions, and the permeability of the membrane did not change significantly within 35 h [99]. In addition, multi-component mixed-matrix membranes were prepared by selecting the polymer PIM-1 with high permeability and the polymer MEEP polyphosphazene with good selectivity as the dispersed phase and adding them to the MOF membrane. As the HKUST-1 reacted with functional groups in the polymer, the CO_2_ permeability of the membrane was enhanced, which reached as high as 25,670 Barrer. Additionally, it was found in adsorption experiments that the balance between the selectivity and permeability of the membrane was broken, and its value exceeded the Robeson upper bound. Its superior performance could effectively reduce the cost of application, and the cost of capturing one ton of CO_2_ using this MOF membrane was as little as $55/tonne [100].

### 4.4. Application in Gas and Solid Phase

The main application of MOF in solid–gas systems is the adsorption of solid particles in the air. With the rapid development of industry, the problem of air pollution has gradually become serious, and millions of people die every year from diseases caused by air pollution. Among them, particulate matter (PM) is regarded as the most harmful powder [101]. Therefore, there is an urgent need to develop a high-efficiency filtration membrane that can filter particles in the air.

A MOF membrane that could effectively filter airborne particles has been developed. The membrane was developed with the use of two plastic syringes for simultaneous electrospinning, and its unique biocomponent effectively improved the mechanical properties and tensile strength (3.79 ± 0.12 MPa) of the MOF membrane. In addition, the surface area of the MOF membrane was increased, which caused the PM capture efficiency of the membrane to be improved. In the capture experiments, the removal rates of PM_2.5_ and PM10 reached as high as 90% and 98%, respectively [102]. Additionally, one previous study confirmed that a MOF membrane with excellent adsorption of air particles could be prepared via the in situ growth of ZIF-67 on SiO_2_ nanofibrous membranes. Taking advantage of the structural advantages of the nanofiber membrane, the membrane could not only adsorb solid particles in the air but also adsorb toxic gases such as SO_2_. Moreover, since ZIF-67 was grown in situ rather than doped in the membrane, the contact area between MOF particles and the adsorbate could be greatly increased. The filtering efficiency of the membrane could reach 98.9% under the PM_2.5_ concentration of 1000 μg·m^−3^. Additionally, the SO_2_ adsorption capacity could reach 1234 g/mg [103].

## 5. Conclusions

Compared with traditional polymer membranes or molecular sieve membranes, MOF membranes have more excellent properties, such as large specific surface areas and adjustable pore sizes. In this article, several typical MOF membrane preparation methods are reviewed, including the in situ growth method, secondary growth method, and electrochemical method. In situ, the growth method can obtain high-quality single crystals and the preparation method is simple; the secondary growth method can control the final orientation of the membrane, resulting in denser filtration membranes; whereas the electrochemical method only requires a lower synthesis temperature. In addition, this article compares and analyzes the MOF materials commonly used in the preparation of mixed-matrix membranes, including ZIF, UIO, and HKUST. Due to the good stability and gas adsorption capacity of ZIF, it is currently regarded as the most commonly used filling material for mixed-matrix membranes. In addition, this article also summarizes the specific applications of MOF membranes in different fields. Because of the excellent filtration of the MOF membrane, it has broad application prospects in the fields of seawater desalination and water purification and can effectively remove heavy metals and organic pollutants from industrial wastewater; it can also effectively reduce the shuttle effect of lithium–sulfur batteries through use as a lithium–sulfur battery separator. Its special pore size and porous structure enables it to effectively capture CO_2_, thus showing a good application prospect in controlling the greenhouse effect. Although MOF is widely used at present, it cannot be used on a large scale, and the production costs of MOF membranes are also high; these form the problems encountered in the process of MOF membrane development. Therefore, the selection of MOF membranes prepared using low-cost ligands and the development of MOF membranes that can adapt to industrial conditions are the current issues that research into MOF membranes should address.

## Figures and Tables

**Table 1:** Characteristics of the synthesis methods.

Synthesis Methods	Advantages	Disadvantages	Reference
In situ growth method	Easy to prepare	High energy consumption	[39]
Secondary growth method	Can produce a more dense and continuous membrane	A complex process, difficult to produce on a large scale	[34]
Electrochemical method	Easy to prepare and control membrane structure by altering the voltage	It is necessary to ensure continuous contact between the metallic pattern and the power	[37,39]

**Table 2:** Characteristics of common MOF particles.

Type of Filler	Crystal Structure	Metal Ion	Advantage	Disadvantage	Reference
ZIF	Tetrahedral network structure	Zn/Co	Thermally and chemically stable	Expensive	[29,41,42,43]
UIO	Three-dimensional microporous structure	Zr	Thermal stability, water stability, good selectivity to gases	Certain degrees of ligand defects	[44,45,46,47]
MIL	Open face-centered cubic microporous framework	Al/Cr/Fe	High stability, large specific surface area	High cost	[48,49]

**Table 3:** Mixed-matrix membranes with UIO-66 in different combinations.

Filler	Polymer	CO_2_/N_2_ Selectivity	CO_2_/CH_4_ Selectivity	Loading (wt %)	References
UiO-66-NH_2_@ICA	Matrimid		64.7	10	[62]
Azo-UiO-66	Matrimid	40		20	[65]
UiO-66-PEI @pSBMA	6FDA-ODA		60.32	15	[66]
UiO-66-PEI	6FDA-ODA		56.49	15	[63]
UiO-66-NH_2_	6FDA-ODA		51.6	25	[67]
	PES	50.2		15	[61]
UiO-66-Br	ODPA-TMPDA	34.5		20	[68]
UiO-66-(OH)_2_	ODPA-TMPDA	38.9		20	[68]
UiO-66@HNT	Pebax-1657	76.26		20	[69]

**Table 4:** Application of MOF membranes.

Scope of Application	Effects	References
Lithium–sulfur battery separator	Inhibit the shuttle effect of polysulfides	[8,9]
Industrial waste	Adsorption of heavy metals and pollutants in wastewater	[12,13,14,15]
Seawater	Adsorption of salt ions in seawater	[10,11]

## Data Availability

The data are available from the corresponding author upon reasonable request.
